# Supplementary material for: Predicting visual function by interpreting a neuronal wiring diagram
Source: Nature. 2024 Oct 2;634(8032):113–23. doi: 10.1038/s41586-024-07953-5 (PMC11446822; doi:10.1038/s41586-024-07953-5)
Supplement: Supplementary file 6 — CRF and ERF predictions for individual TmY4 and TmY9 cells. Analogous to Supplementary Data 3, but for TmY target types. Shown are the top four monosynaptic pathways, the strongest pathway passing through each of the top ten intermediary types (ranking from Extended Data Fig. 7), and the trisynaptic pathway Tm1–TmY–Dm3–TmY (see the section entitled Prediction of spatial normalization). [file 41586_2024_7953_MOESM6_ESM.zip › DataS4/TmY9qΓèÑ/720575940622198298.pdf]

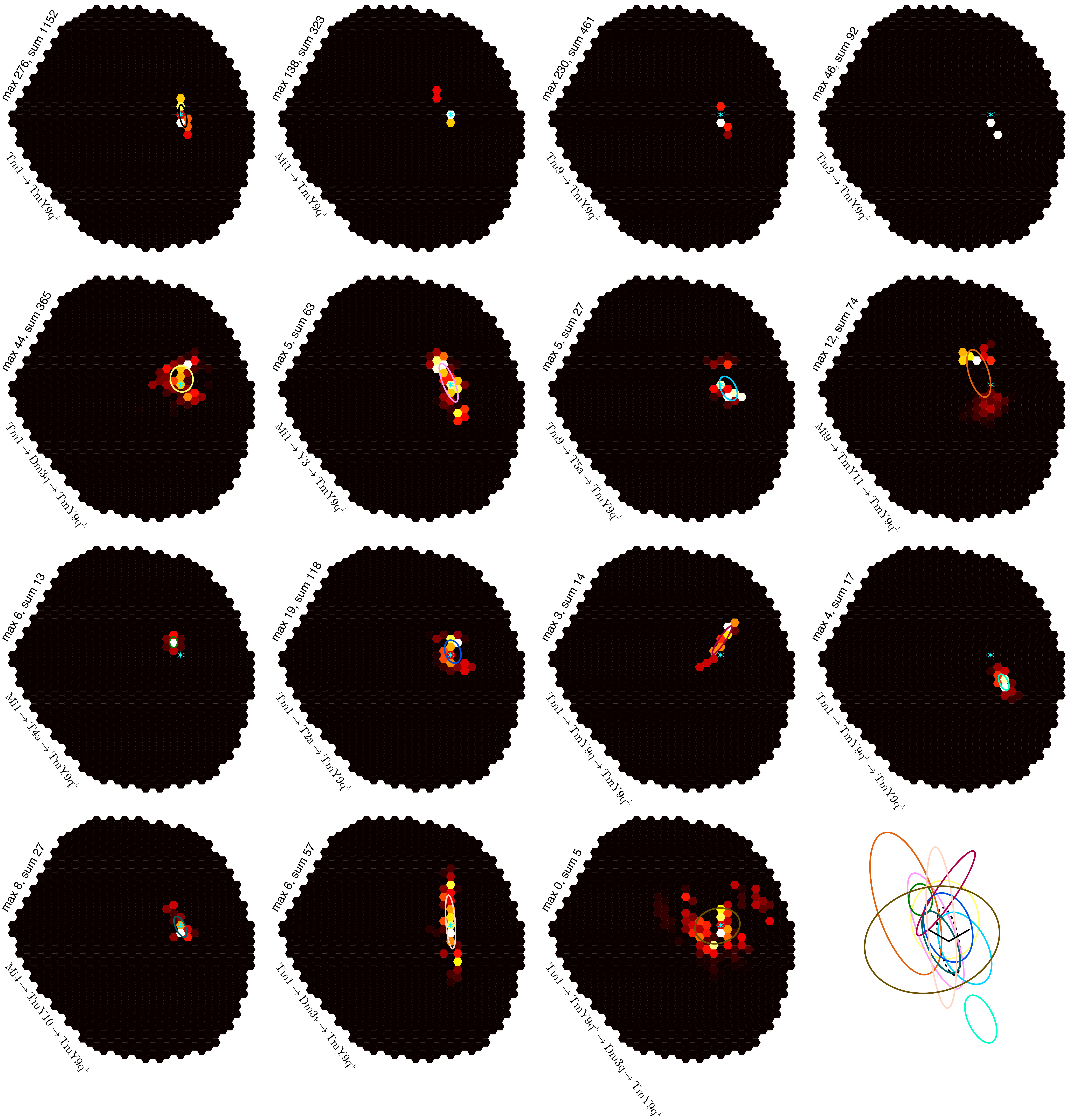
max 276, sum 1152
Tm1 → TmY9q⊥
max 138, sum 323
Mi1 → TmY9q⊥
max 230, sum 461
Tm9 → TmY9q⊥
max 46, sum 92
Tm2 → TmY9q⊥
max 44, sum 365
Tm1 → Dm3q → TmY9q⊥
max 5, sum 63
Mi1 → Y3 → TmY9q⊥
max 5, sum 27
Tm9 → T5a → TmY9q⊥
max 12, sum 74
Mi9 → TmY11 → TmY9q⊥
max 6, sum 13
Mi1 → T4a → TmY9q⊥
max 19, sum 118
Tm1 → T2a → TmY9q⊥
max 3, sum 14
Tm1 → TmY9q → TmY9q⊥
max 4, sum 17
Tm1 → TmY9q⊥ → TmY9q⊥
max 8, sum 27
Mi4 → TmY10 → TmY9q⊥
max 6, sum 57
Tm1 → Dm3v → TmY9q⊥
max 0, sum 5
Tm1 → TmY9q⊥ → Dm3q → TmY9q⊥
